# Survival after extracorporeal membrane oxygenation in severe COVID-19 ARDS: results from an international multicenter registry

**DOI:** 10.1186/s13054-021-03486-9

**Published:** 2021-03-01

**Authors:** Alexander Supady, Fabio Silvio Taccone, Philipp M. Lepper, Stephan Ziegeler, Dawid L. Staudacher, for the COVEC-Study Group, Alexander Supady, Fabio Silvio Taccone, Philipp M. Lepper, Stephan Ziegeler, Dawid L. Staudacher, Jeff DellaVolpe, Dominik Scharpf, Matthias Ulmer, Maximilian Halbe, Alexander Vogt, Raj Ramanan, David Boldt, Stephanie-Susanne Stecher, Andrea Montisci, Tobias Spangenberg, Olivier Marggraf, Chandra Kunavarapu, Lorenzo Peluso, Sebastian Muenz, Monica Buerle, Naveen G. Nagaraj, Sebastian Nuding, Catalin Toma, Vadim Gudzenko, Hans Joachim Stemmler, Federico Pappalardo, Georg Trummer, Christoph Benk, Guido Michels, Christoph Bode, Daniel Duerschmied, Constantin von zur Muehlen, Klaus Kaier, Daniel Brodie, Tobias Wengenmayer

**Affiliations:** 1 Department of Medicine III (Interdisciplinary Medical Intensive Care), Medical Center, Faculty of Medicine, University of Freiburg, Hugstetter Strasse 55, 79106 Freiburg, Germany; 2 Department of Cardiology and Angiology I, Heart Center, University of Freiburg, Freiburg, Germany; 3 Heidelberg Institute of Global Health, University of Heidelberg, Heidelberg, Germany; 4 Department of Intensive Care, Erasme Hospital, Université Libre de Bruxelles, Brussels, Belgium; 5 Department of Internal Medicine V – Pneumology, Allergology and Critical Care Medicine, Saarland University Medical Center and University of Saarland, Homburg, Germany; 6 Department of Anesthesiology, Intensive Care Medicine and Pain Management, Hospital Ibbenbueren, Ibbenbueren, Germany

Survival of coronavirus disease 2019 (COVID-19) patients with severe respiratory failure treated with veno-venous extracorporeal membrane oxygenation (V-V ECMO) ranges around 60%, according to recent studies [1, 2]. Initial recommendations for the use of V-V ECMO in COVID-19-related acute respiratory distress syndrome (ARDS) were largely based on studies from the pre-COVID-19 era [3, 4]. V-V ECMO was initiated in younger patients (i.e., < 71 years) and in those with rather short duration of mechanical ventilation (MV) prior to ECMO (i.e., < 7 or < 11 days, respectively) [1, 5]. While it is reasonable to focus on selected ECMO cohorts in controlled trials, survival of COVID-19 patients treated with ECMO beyond these limitations remains unclear, so far. Here, we report survival data of COVID-19 ARDS patients treated with V-V ECMO from a large, international multicenter registry.

Data were collected retrospectively from medical records at 3 ECMO centers in the USA, 9 in Germany, and 1 in Switzerland, Belgium, and Italy. At the participating centers, all patients with reverse transcriptase polymerase chain reaction (rtPCR) positive testing for SARS-CoV-2, who received V-V ECMO from March 12 to June 5, 2020 (i.e., during the first wave of the pandemic), were included.

A total of 127 patients were analyzed: 53/127 (41.7%) of them survived at day 90 after ECMO implantation (Table 1). Higher survival was observed in patients younger than 71 years when compared to others (Fig. 1: 110/127, 45.5% vs. 17/127, 17.6%, *p* = 0.004). However, patients being on MV before ECMO for less than 7 days had slightly higher survival rate than those with longer MV course though not reaching statistical significance (77/127, 46.8% vs. 50/127, 34.0%; *p* = 0.167). Similar results were observed when the duration of MV was dichotomized in < 11 and ≥ 11 days (101/127, 45.5% vs. 26/127, 26.9%; *p* = 0.044).

**Table 1:** Patient baseline characteristics before initiation of extracorporeal membrane oxygenation (ECMO) and 90-day survival

	Total	Mechanical ventilation prior to ECMO < 7 days (n = 77)	Mechanical ventilation prior to ECMO ≥ 7 days (n = 50)	Mechanical ventilation prior to ECMO < 11 days (n = 101)	Mechanical ventilation prior to ECMO ≥ 11 days (n = 26)	Age < 71 years (n = 110)	Age ≥ 71 years (n = 17)
Number of patients, No. (% of total)	127 (100)	77 (61)	50 (39)	101 (80)	26 (20)	110 (87)	17 (13)
Female gender, No. (%)	27 (21)	20 (26)	7 (14)	22 (22)	5 (19)	24 (22)	3 (18)
90-day survival, No. (%)	53 (41.7)	36 (46.8)	17 (34.0)	46 (45.5)	7 (26.9)	50 (45.5)	3 (17.6)
Age [years], median (IQR)	59.0 (53.0–66.0)	57.0 (48.5–64.5)	61.0 (56.0–69.0)	58.0 (51.0–66.0)	61.0 (55.8–69.3)	58.0 (51.0–64.0)	73.0 (72.5–75.5)
Duration of invasive mechanical ventilation before ECMO [days], median (IQR)	5 (2–9)	2 (1–4)	11 (8–15)	3 (1–6)	15 (12–20.5)	5 (1–9)	6 (3.5–9.5)
SOFA, median (IQR)	9.0 (7.0–10.0)	8.0 (6.5–10.0)	9.0 (8.0–10.25)	9.0 (7.0–10.0)	9.0 (8.0–10.3)	9.0 (7.0–10.0)	10.0 (8.5–10.0)

All statistical analyses were performed using GraphPad Prism 9 (GraphPad Software, San Diego, USA)

*IQR* interquartile range, *SOFA* Sequential Organ Failure Assessment

**Fig. 1:**
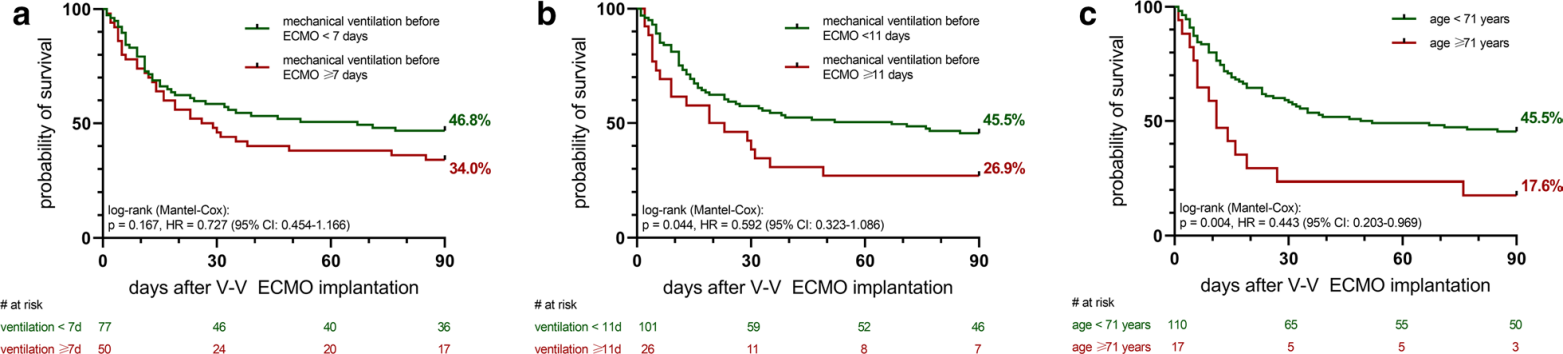
Kaplan–Meier curves for **a** survival of patients on mechanical ventilation < 7 days or ≥ 7 days before V-V ECMO (Cox proportional hazards model including SOFA score: *p* = 0.215, HR 0.755 (95% CI 0.484–1.178), **b** survival of patients on mechanical ventilation < 11 days or ≥ 11 days before V-V ECMO (Cox proportional hazards model including SOFA score: *p* = 0.052, HR 0.604 (95% CI 0.363–1.005), **c** survival of patients aged < 71 years or ≥ 71 years before V-V ECMO (Cox proportional hazards model including SOFA score: *p* = 0.008, HR 0.464 (95% CI 0.263–0.820). All statistical analyses were performed using GraphPad Prism 9 (GraphPad Software, San Diego, USA) and SPSS 27 (IBM, Armonk, New York, USA). *V-V ECMO* veno-venous extracorporeal membrane oxygenation

Our findings derive from an international multicenter registry of COVID-19-related ARDS patients treated with V-V ECMO. 90-day survival in our cohort was 41.7%, which was lower than previously described for COVID-19 patients treated with V-V ECMO in large registries and survival reported for non-COVID-19 ARDS patients [1, 2, 5]. The lower survival rate might be attributable to a more liberal use of V-V ECMO in this real-world cohort outside a prospective trial or to a different policy than in other ECMO centers. Even though survival of patients treated with ECMO even after longer periods of time of MV was lower than survival of patients with early initiation of ECMO, the latter still showed considerable survival rates. Our results therefore challenge strict contraindications for initiation of ECMO in COVID-19 patients solely based on duration of MV. Moreover, even though 90-day-survival of patients aged ≥ 71 years was significantly lower than for patients < 71 years, not all treatments in this elderly population ended fatal. Therefore, age limits should be viewed with caution and decisions for or against the use of ECMO for patients above 70 years of age should be performed on an individual case-by-case level.

The main strength of our study is the high number of patients and multicenter analysis. However, our results are limited due to the retrospective design, small case volume at each center, the lack of a control group, and potential differences in ECMO practices and criteria for ECMO at the different centers.

In conclusion, our data may support the use of V-V ECMO in severe COVID-19 ARDS, also after prolonged periods of mechanical ventilation in selected patients. Upper age limits should be viewed with caution and not taken as the sole reason to withhold ECMO treatment.

## Data Availability

All data will be available from the corresponding author on reasonable request.
